# Studies of mice deleted for *Sox3* and *uc482*: relevance to X-linked hypoparathyroidism

**DOI:** 10.1530/EC-19-0478

**Published:** 2020-01-20

**Authors:** Katherine U Gaynor, Irina V Grigorieva, Samantha M Mirczuk, Sian E Piret, Kreepa G Kooblall, Mark Stevenson, Karine Rizzoti, Michael R Bowl, M Andrew Nesbit, Paul T Christie, William D Fraser, Tertius Hough, Michael P Whyte, Robin Lovell-Badge, Rajesh V Thakker

**Affiliations:** 1 Academic Endocrine Unit, Radcliffe Department of Medicine, University of Oxford, Oxford Centre for Diabetes, Endocrinology and Metabolism, Churchill Hospital, Oxford, UK; 2 The Francis Crick Institute, London, UK; 3 Norwich Medical School, Faculty of Medicine and Health Sciences, University of East Anglia, Norwich, UK; 4 MRC Mammalian Genetics Unit, MRC Harwell Institute, Harwell Science and Innovation Campus, Oxfordshire, UK; 5 Washington University in St Louis School of Medicine, Center for Metabolic Bone Disease and Molecular Research, St Louis, Missouri, USA

**Keywords:** parathyroid-related disorders, transcription factors, genetic research, genetic animal models, preclinical studies

## Abstract

Hypoparathyroidism is genetically heterogeneous and characterized by low plasma calcium and parathyroid hormone (PTH) concentrations. X-linked hypoparathyroidism (XLHPT) in two American families is associated with interstitial deletion-insertions involving deletions of chromosome Xq27.1 downstream of *SOX3* and insertions of predominantly non-coding DNA from chromosome 2p25.3. These could result in loss, gain, or movement of regulatory elements, which include ultraconserved element *uc482*, which could alter *SOX3* expression. To investigate this, we analysed *SOX3* expression in EBV-transformed lymphoblastoid cells from three affected males, three unaffected males, and four carrier females from one XLHPT family. SOX3 expression was similar in all individuals, indicating that the spatiotemporal effect of the interstitial deletion-insertion on SOX3 expression postulated to occur in developing parathyroids did not manifest in lymphoblastoids. Expression of *SNTG2*, which is duplicated and inserted into the X chromosome, and *ATP11C*, which is moved telomerically, were also similarly expressed in all individuals. Investigation of male hemizygous (*Sox3*^−/Y^ and *uc482*^−/Y^) and female heterozygous (*Sox3*^+/^^−^ and *uc482*^+/^^−^) knockout mice, together with wild-type littermates (male *Sox3*^+/Y^ and *uc482*^+/Y^, and female *Sox3*^+/+^ and *uc482*^+/+^), revealed *Sox3*^−/Y^, *Sox3*^+/^^−^, *uc482*^−^/Y, and *uc482*^+/^^−^ mice to have normal plasma biochemistry, compared to their respective wild-type littermates. When challenged with a low calcium diet, all mice had hypocalcaemia, and elevated plasma PTH concentrations and alkaline phosphatase activities, and *Sox3*^−/Y^, *Sox3*^+/^^−^, *uc482*^−/Y^, and *uc482*^+/^^−^ mice had similar plasma biochemistry, compared to wild-type littermates. Thus, these results indicate that absence of *Sox3* or *uc482* does not cause hypoparathyroidism and that XLHPT likely reflects a more complex mechanism.

## Introduction

Heritable hypoparathyroidism (HPT) is a genetically heterogeneous disease, characterized biochemically by hypocalcaemia, hyperphosphatemia, low plasma parathyroid hormone (PTH) concentrations and inappropriately normal or high urinary calcium excretion (1). Genetic abnormalities causing HPT may lead to complex congenital syndromes or to an isolated endocrinopathy, for which autosomal dominant, autosomal recessive and X-linked modes of inheritance have been described (OMIM #146200 and %307700) (2). These genetic abnormalities may result in defects in PTH itself, parathyroid gland development, or parathyroid gland function. For example, mutations in *PTH* cause a lack of functional PTH protein (3), mutations in the parathyroid-specific transcription factor glial cells missing B (*GCMB*) result in defective parathyroid gland development (4, 5), and gain-of-function mutations in the calcium-sensing receptor (CaSR) may suppress parathyroid gland function in association with hypocalcaemia (6).

**Figure 1:**
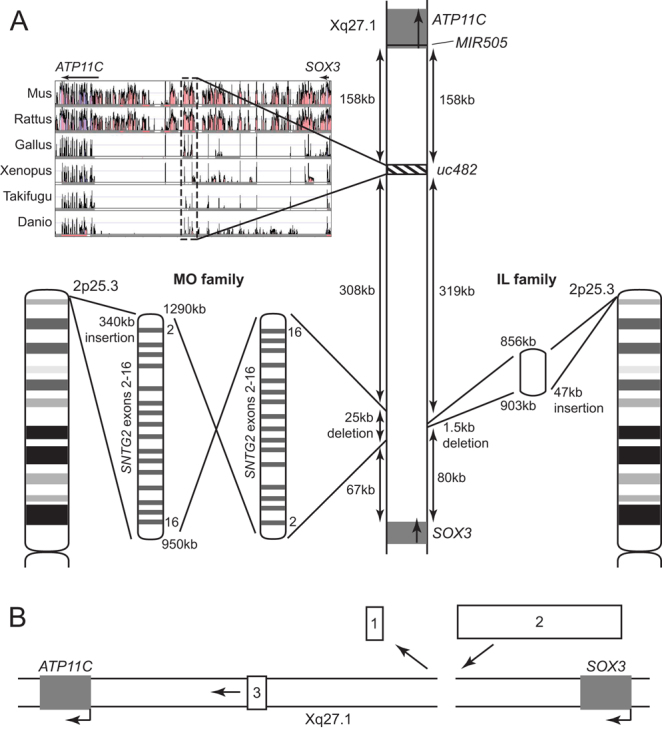
Chromosomal rearrangements associated with XLHPT. (A) Unrelated families from Missouri (MO) and Illinois (IL), USA, have interstitial deletion-insertions involving chromosome Xq27.1 and chromosome 2p25.3. In the MO kindred, a 25 kb Xq27.1 deletion is replaced by a ~340 kb inverted insertion containing exons 2–16 of syntrophin gamma 2 (*SNTG2*). In the IL family, a 1.5 kb Xq27.1 deletion is replaced by a 47 kb 2p25.3 insertion that does not contain any coding DNA. These deletion-insertions occur downstream of sex-determining region Y (SRY)-box 3 (*SOX3*) and upstream of adenosine triphosphatase 11C (*ATP11C*) and the ultraconserved element *uc482*. *uc482* is a genome region conserved from human through mouse (Mus), rat (Rattus), chicken (Gallus), frog (Xenopus), and puffer fish (Takifugu) to zebrafish (Danio) (plotted using Vista Browser). *MIR505* (chromosomal location 139,924,148-139,924,231 (GRCh38/hg38)) is located within the first intron in two of the nine *ATP11C* (chromosomal location 139,726,346-139,945,276 (GRCh38/hg38)) transcripts and upstream of the remaining seven transcripts, five of which are protein-coding. (B) Three possible mechanisms by which the XLHPT deletion-insertions may alter *SOX3* expression are (1) loss of an enhancer or repressor (represented by box 1) from Xq27.1; (2) gain of an enhancer or repressor (represented by box 2) from 2p25.3; or (3) movement of an enhancer or repressor (represented by box 3) away from *SOX3*, as the insertion is larger than the deletion.

X-linked HPT (XLHPT, OMIM %307700) was first reported in 1960 (7) in a large kindred from Missouri (MO), USA and subsequently in a related family also from MO (8, 9, 10), and more recently in a third, unrelated family from Illinois (IL), USA (11). Both families from MO and the family from IL had interstitial deletion-insertions involving deletion of a non-coding region on chromosome Xq27.1 between sex-determining region Y (SRY)-box 3 (*SOX3*) and adenosine triphosphatase 11C (*ATP11C*), and an insertion of a larger section emanating from chromosome 2p25.3 (8, 11) (Fig. 1A). In the MO kindred, this consisted of a ~25 kb deletion of non-coding DNA from Xq27.1 ~67 kb downstream of *SOX3* and an inverted insertion of ~340 kb containing exons 2–16 of syntrophin gamma 2 (*SNTG2*) from 2p25.3 (8) (Fig. 1A). In the IL family, ~1.5 kb was deleted from Xq27.1 ~80 kb downstream of *SOX3* and ~47 kb of non-coding DNA was inserted from 2p25.3, which differed from the inserted DNA in the MO kindred (11) (Fig. 1A). Although the 340 kb insertion in the MO kindred contained 15 exons of *SNTG2*, no open reading frames were present (8), and the 47 kb insertion in the IL family did not contain coding DNA. Similarly, neither of the X chromosome deletions removed coding DNA. We therefore hypothesized that these rearrangements may alter the function of a regulatory element or nearby gene, that affects *SOX3* expression, which has been demonstrated in developing mouse parathyroids between embryonic day (E)10.5 and E15.5 (8). *SOX3* expression was reported to be altered by a large 774 kb insertion downstream of *SOX3* in patients with XX male sex reversal, demonstrating that *SOX3* expression may be susceptible to position effects, similar to those reported for *SOX9* and *SRY* (12, 13), although *SOX3*-coding mutations have not been identified in male patients with HPT (14).

Thus, in the XLHPT families there are three possible mechanisms that could result in such alterations in *SOX3* expression (Fig. 1B). Firstly, deletions of X chromosome sequences may result in loss of a repressor or enhancer of *SOX3* expression. Secondly, insertions of chromosome 2 sequences may result in insertion of DNA that may repress or enhance *SOX3* expression. Thirdly, deletion-insertions, that result in a net gain of DNA on the X chromosome, may move a repressor or enhancer of *SOX3* expression further away from *SOX3*, thereby altering its effect on *SOX3* expression, that is, a position effect (Fig. 1B). Gene enhancers and repressors are often evolutionarily conserved, and >60% of ultraconserved (uc) non-coding elements that are conserved in human through to *Takifugu rubripes* (puffer fish), may act as positive enhancers (15). One such element, designated *uc482* (or highly conserved non-coding element (HCNE) 8), is located ~400 kb downstream of *SOX3* and was reported to be a likely enhancer of *Sox3* expression in *Danio rerio* (zebrafish) (16). Furthermore, *uc482* is moved further away from *SOX3* by both of the deletion-insertions causing XLHPT (Fig. 1B).

To explore the possible roles of *SOX3* and *uc482* in HPT, we undertook studies to determine the effects of the interstitial deletion-insertion on *SOX3* expression in patients with XLHPT and in knock-out mice lacking alleles of *Sox3* and *uc482*.

## Materials and methods

### Human studies

Venous blood samples were collected after informed consent from individuals and following protocols approved by local and national ethics committees (Multi-centre Research Ethics Committee (London, UK; MREC 02/2/93) and the Human Research Protections Office, Washington University School of Medicine, St. Louis, MO, USA).

### DNA sequence analysis

DNA was extracted from leukocytes using the Gentra Puregene blood kit (Qiagen) and standard protocols. PCR amplification of DNA across the deletion-insertion boundaries was carried out using primers previously described (8), and Sanger DNA sequence analysis was performed using the BigDye Terminator v3.1 Cycle Sequencing Kit (Life Technologies) and an ABI automated capillary sequencer (Applied Biosystems), as described (4, 8).

### Quantitative real-time PCR (qRT-PCR)

Lymphocytes were Epstein-Barr virus (EBV)-transformed to generate a lymphoblastoid cell line as previously described (3), and mRNA extracted using the Oligotex mRNA mini kit (Qiagen) and standard protocols. cDNA was generated using Quantiscript reverse transcriptase (Qiagen), and quantitative reverse transcriptase (qRT)-PCRs performed using the QuantiTect SYBR Green PCR kit (Qiagen) in quadruplicate for each individual, and *SOX3*, *SNTG2*, *ATP11C*, and *GAPDH* specific primers, using a Rotorgene 5 (Qiagen), as described (17). Expression of genes was normalized to *GAPDH* expression and analyzed by the comparative ΔΔC_T_ method (18).

### Mouse studies

All animal studies were approved by the University of Oxford Ethical Review Committee and were licensed (project license numbers PPL 30/2241, PPL 30/2739 and PPL 30/3251) under the Animals (Scientific Procedures) Act 1986, issued by the UK Government Home Office. *Sox3*-knockout mice were maintained on the MF1 outbred background (19) and *uc482*-knockout mice were maintained on the C57Bl/6 background (20). Mice were genotyped using DNA extracted from ear biopsies followed by PCR assay. PCR primers for *Sox3* genotyping were WT F: 5′-TCGGGTGGTGGGGAAGGGGTTAT-3′, WT R: 5′-GTGGGGTGTGCGGCTCAGGTAG-3′, KO F: 5′-CACGGCGAGCCTGTCAATCACGAG-3′, KO R: 5′-TTGATGCCGTTCTTTTGCTTGTCG-3′; and for *uc482* genotyping were For: 5′-GGAAATGAGGCCGAGTCAAG-3′; WT R: 5′-TACGAAGACATGTACCTGTGCG-3′; KO R: 5′-TGGACTTGTCAGCTTCTTCCAA-3′. After weaning at age 21 days, mice were fed either a synthetic control diet (Dyets Inc., Bethlehem, PA, USA) containing 0.95% calcium and 4.50 iU/g vitamin D_3_ or a synthetic low-calcium diet containing 0.001% calcium and 0.0 iU/g vitamin D_3_ (Dyets Inc.), as previously described (21). The low calcium/no vitamin D_3_ diet has previously been shown to unmask defects in the ability of the parathyroid glands to respond to deficiencies in dietary calcium, which may not be evident when calcium is abundant (21). Food and water were allowed *ad libitum*. Mice were killed and blood was collected from the jugular vein for plasma analysis using an AU400 clinical chemistry analyser (Olympus), and plasma calcium was corrected for albumin (Corr. Ca) using the formula: Corr. Ca = Ca (mmol/L) − ((Alb (g/L) − 30) × 0.017), as described previously (22). PTH concentrations were measured using an ELISA for mouse intact PTH (Immutopics, Quidel, San Diego, CA, USA), as described previously (22).

### Histological studies

To study parathyroids of the knockout mice, the trachea, thyroid, and parathyroids were dissected *en bloc* and fixed for 24 h in 10% neutral buffered formalin. Paraffin sections (6 µm thickness) were dewaxed and stained with haematoxylin and eosin (H&E) using standard techniques. H&E stained sections were viewed using an Eclipse E400 light microscope (Nikon) and images acquired using NIS-elements BR2.30 imaging software (Nikon). An estimation of parathyroid gland size was performed by measuring the largest cross-sectional area of the parathyroid gland in serial sections from each mouse, as described previously (21, 23), and corrected for body weight.

### Statistical analyses

For human qRT-PCR analyses, data were analyzed using one-way ANOVA, and all data are represented as mean ± s.d. fold change relative to unaffected males. For animal studies, data were analyzed using unpaired Student’s *t*-test. All data are represented as mean ± s.d., and for plasma biochemical results, significances are reported after Bonferroni correction for multiple comparisons.

## Results

### Expression of *SOX3*, *ATP11C* and *SNTG2* in patients with XLHPT

**Figure 2:**
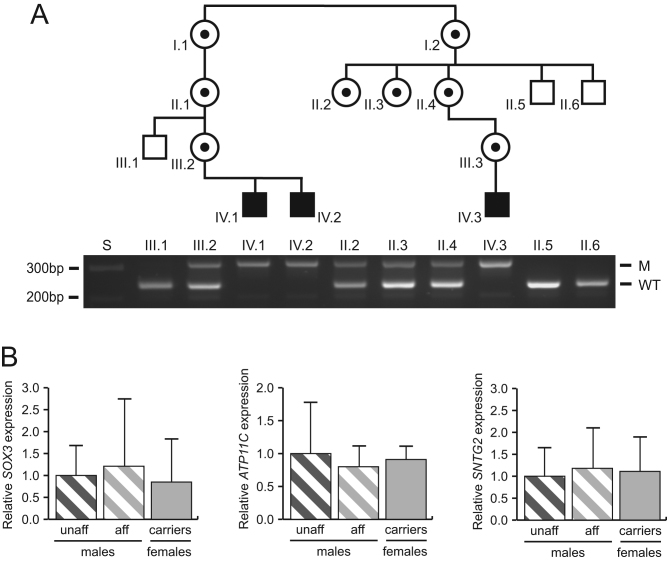
*SOX3*, *SNTG2*, and *ATP11C* expression in XLHPT family members. (A) Confirmation of the Xq27.1-2p25.3 deletion-insertion by PCR using primers spanning the telomeric breakpoint (Fig. 1) in four carrier females (circles with dots), and three affected males (filled squares) from the MO kindred. Three unaffected related males (open squares) in whom the Xq27.1-2p25.3 insert is not present are also shown. The PCR primers were designed to yield a wild-type (WT) product of 237 bp in size, and a mutant (M) product of 321 bp in size. S = size marker. (B) Quantitative reverse transcriptase (qRT)-PCR analyses of *SOX3*, *ATP11C*, and *SNTG2* expression using mRNA obtained from EBV-lymphoblastoids from unaffected males (unaff) (*n* = 3), affected males (aff) (*n* = 3) and carrier females (carriers) (*n* = 4), (*n* = 4 replicates for each individual). Data are displayed as mean + s.d. relative to expression in unaffected males.

PCR analysis using primers, previously described (8), that spanned the telomeric breakpoint, was used to confirm the presence or absence of the X chromosome deletion-insertion in three affected males, four carrier females, and three unaffected related males from the MO kindred (Fig. 2A). Thus, individuals II.5, II.6 and III.1, who are unaffected males with normocalcaemia, were confirmed to have only the wild-type (normal) allele; individuals IV.1, IV.2 and IV.3, who are affected males with hypocalcaemia due to XLHPT, were confirmed to have only the mutant allele; and individuals II.2, II.3, II.4 and III.2, who are carrier females with normocalcaemia, were confirmed to be heterozygous in having wild-type and mutant alleles (Fig. 2A). Parathyroid glands from XLHPT patients (7), and normal individuals were not available to study the expression of *SOX3* and *ATP11C*, which flank the interstitial deletion-insertion involving chromosomes Xq27.1 and 2p25.3, and the inserted region of *SNTG2* in the XLHPT MO kindred (Fig. 1). Lymphoblastoid cell mRNA was therefore used to undertake qRT-PCR analysis of *SOX3*, *ATP11C* and *SNTG2* expression (Fig. 2B). *SOX3* expression (mean ± s.d,) was similar in affected males (1.21 ± 1.53-fold) and carrier females (0.85 ± 0.98-fold), when compared to unaffected males (1.00 ± 0.68-fold; *P* > 0.8) (Fig. 2B). qRT-PCR analyses of *ATP11C*, which is located downstream of the deletion-insertion, and *SNTG2*, of which exons 2–16 are inserted from chromosome 2p25.3, revealed no significant differences in expression of *ATP11C* or *SNTG2* between affected males (0.80 ± 0.32-fold and 1.18 ± 0.92-fold, respectively) or carrier females (0.91 ± 0.20-fold and 1.11 ± 0.79-fold, respectively), when compared to unaffected males (1.00 ± 0.78-fold and 1.00 ± 0.65-fold; *P* > 0.8) (Fig. 2B). These results indicated that specific spatiotemporal effects of the XLHPT-associated interstitial deletion-insertions on expression of *SOX3* or other genes, *ATP11C* and *SNTG2*, postulated to occur in the developing parathyroids, were not occurring in lymphoblastoid cells, which are not representative of parathyroid cells. We therefore studied mouse knockout models deleted for *Sox3* and *uc482*.

### Mice deleted for* Sox3* have normal plasma calcium and PTH concentrations

**Figure 3:**
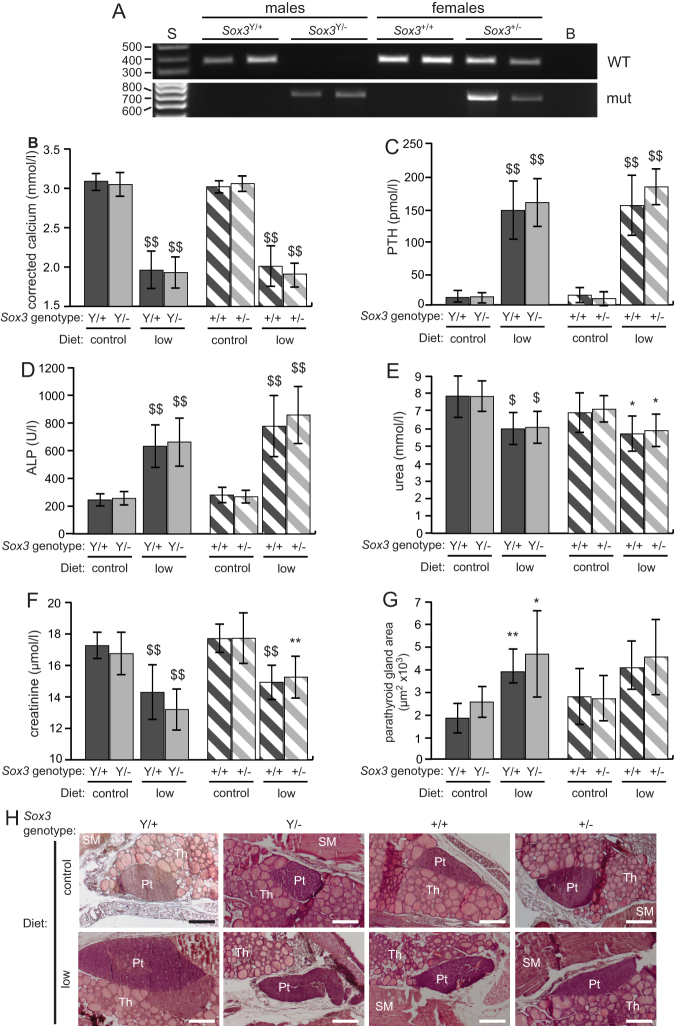
Plasma biochemistry and parathyroid gland sizes in wild-type and mutant *Sox3* mice consuming either control or low calcium and vitamin D diets. (A) Genotyping of wild-type (*Sox3*^+/Y^) and hemizygous (*Sox3*^−/Y^) male, and wild-type (*Sox3*^+/+^) and heterozygous (*Sox3*^+/^^−^) female mice. S: size marker; B: blank; WT: wild-type band; mut: mutant band. (B) Plasma corrected calcium; (C) plasma PTH; (D) plasma alkaline phosphatase (ALP) activity; (E) plasma urea; (F) plasma creatinine; (G) parathyroid gland area. Wild-type males (+/Y) and mutant males (−/Y) are shown by the dark grey and light grey solid bars, respectively. Wild-type females (+/+) and mutant carrier females (+/−) are shown by the dark grey and light grey hatched bars, respectively. Data are displayed as mean ± s.d. *P < 0.05; **P < 0.01; ^$^*P* < 0.001; ^$$^*P* < 0.0001 for mice on the low calcium and vitamin D (low Ca^2+^ and vit D) diet compared to mice of the same genotype on the normal diet. (H) Representative histological images used for quantification of parathyroid gland area. Pt: parathyroid; Th: thyroid; SM: strap muscle. Scale bars = 200 µm.

Mice were genotyped (Fig. 3A), and wild-type (*Sox3*^+/Y^) and hemizygous (*Sox3*^−/Y^) male adult mice, and wild-type (*Sox3*^+/+^) and heterozygous (*Sox3*^+/^^−^) female adult mice were found to have normal body weights and plasma biochemistry at ages 6 and 28 weeks (Table 1). Thus, corrected calcium concentrations in plasma were similar in: *Sox3*^+/Y^ mice compared to *Sox3*^-/Y^ mice (3.08 ± 0.11 mmol/L vs 3.05 ± 0.15 mmol/L at 6 weeks, and 2.83 ± 0.19 mmol/L vs 2.79 ± 0.12 mmol/L at 28 weeks, *n* = 10–21); and in *Sox3*^+/+^ mice compared to *Sox3*^+/^^–^ mice (3.02 ± 0.08 mmol/L vs 3.06 ± 0.10 mmol/L at 6 weeks, and 2.65 ± 0.12 mmol/L vs 2.73 ± 0.11 mmol/L at 28 weeks, *n* = 9–25). Plasma PTH concentrations were also similar in: *Sox3*^+/Y^ and *Sox3*^−/Y^ mice (11.8 ± 7.6 pmol/L vs 12.8 ± 10.4 pmol/L at 6 weeks, and 41.8 ± 31.4 pmol/L vs 35.9 ± 34.1 pmol/L at 28 weeks, *n* = 14–22); and in *Sox3*^+/+^ and *Sox3*^+/^^−^ mice (15.3 ± 11.8 pmol/L vs 9.9 ± 10.6 pmol/L at 6 weeks, and 35.6 ± 23.8 pmol/L vs 24.8 ± 19.1 pmol/L at 28 weeks, *n* = 12–24) (Table 1). In addition, the plasma phosphate, urea, creatinine and albumin concentrations, and alkaline phosphatase activity were similar in wild-type and mutant *Sox3* mice at 6 and 28 weeks of age (Table 1).

**Table 1:** Plasma biochemistry in 6- and 28-week-old wild-type and mutant *Sox3* mice consuming the control diet.

Genotype	Males		Females	
	*Sox3*^+/Y^	*Sox3*^−/Y^	*Sox3*^+/+^	*Sox3*^+/−^
Age: 6 weeks	(*n* = 19–22)	(*n* = 14–18)	(*n* = 23–25)	(*n* = 12–15)
Body weight (g)	11.85 ± 3.49	11.74 ± 3.70	12.05 ± 2.30	12.12 ± 2.71
Corr. Ca (mmol/L)	3.08 ± 0.11	3.05 ± 0.15	3.02 ± 0.08	3.06 ± 0.10
PTH (pmol/L)	11.8 ± 7.6	12.8 ± 10.4	15.3 ± 11.8	9.9 ± 10.6
Phosphate (mmol/L)	4.24 ± 0.76	4.10 ± 0.68	4.07 ± 0.62	3.88 ± 0.83
ALP (U/L)	258.7 ± 47.2	270.2 ± 49.4	296.5 ± 58.4	282.4 ± 48.4
Urea (mmol/L)	7.85 ± 1.24	7.83 ± 0.87	6.90 ± 1.13	7.10 ± 0.74
Creatinine (µmol/L)	17.3 ± 0.8	16.8 ± 1.3	17.7 ± 0.9	17.7 ± 1.6
Albumin (g/L)	30.5 ± 1.7	31.6 ± 2.7	31.7 ± 2.0	32.3 ± 1.7
Age: 28 weeks	(*n* = 10–14)	(*n* = 15–19)	(*n* = 8–12)	(*n* = 14–18)
Body weight (g)	47.51 ± 6.81	50.17 ± 9.23	32.16 ± 1.66	40.93 ± 8.92
Corr. Ca (mmol/L)	2.83 ± 0.19	2.79 ± 0.12	2.65 ± 0.12	2.73 ± 0.11
PTH (pmol/L)	41.8 ± 31.4	35.9 ± 34.1	35.6 ± 23.8	24.8 ± 19.1
Phosphate (mmol/L)	3.33 ± 0.49	3.26 ± 0.54	2.97 ± 0.21	3.06 ± 0.64
ALP (U/L)	81 ± 15	74 ± 12	98 ± 24	90 ± 24
Urea (mmol/L)	8.3 ± 1.12	8.2 ± 0.82	7.2 ± 1.4	8.1 ± 1.2
Creatinine (µmol/L)	15.9 ± 0.9	16.2 ± 1.5	16.1 ± 1.7	17.3 ± 1.5
Albumin (g/L)	29.5 ± 2.2	30.7 ± 0.8	32.2 ± 1.5	31.9 ± 1.9

Mean ± s.d, values are shown. Wild-type males (*Sox3*^+/Y^), mutant males (*Sox3*^−/Y^), wild-type females (*Sox3*^+/+^), and mutant carrier females (*Sox3*^+/^^−^). There were no significant differences between knockout and wild-type littermates at the same age.

ALP, alkaline phosphatase; Corr. Ca, corrected calcium; PTH, parathyroid hormone.

The standard control mouse chow contains 0.95% calcium and 4.5 iU/g vitamin D_3_, which represent levels that are ~150-fold and ~30-fold higher, respectively, than those recommended for daily intake in humans (21). Furthermore, these high dietary contents of calcium and vitamin D have been reported to mask hypocalcaemia in mice with a different cause of HPT (21). We therefore challenged *Sox3*^+/Y^, *Sox3*^−/Y^, *Sox3*^+/+^, and *Sox3*^+/^^−^ mice with a diet low in calcium (0.001%) and lacking in vitamin D_3_ (low diet) from weaning until 60 days old. Wild-type male and female mice on the low diet had significantly lower plasma corrected calcium concentrations than those on the control diet (mean ± s.d.) (low diet versus control diet for *Sox3*^+/Y^ = 1.96 ± 0.24 mmol/L vs 3.08 ± 0.11 mmol/L, *P* < 0.001; and *Sox3*^+/+^ = 2.01 ± 0.26 mmol/L vs 3.02 ± 0.08 mmol/L, *P* < 0.001, *n* = 16–25) (Fig. 3B). These lower plasma corrected calcium concentrations, were not due to lower plasma albumin concentrations when on the low diets, as these plasma albumin concentrations were similar (Sox3^+/+^ = 32.70 ± 0.35 g/L, Sox3^+/^^−^ = 32.58 ± 0.24 g/L, Sox3^+/Y^ = 31.50 ± 0.34 g/L and Sox3^−/Y^ = 32.40 ± 0.40 g/L), on low diet to those observed in mice on the control diet (Table 1). This hypocalcaemia was associated with significant increases in plasma PTH concentrations (*Sox3*^+/Y^ = 150.1 ± 46.3 pmol/L vs 11.8 ± 7.6 pmol/L, *P* < 0.001; and *Sox3*^+/+^ = 157.8 ± 47.6 pmol/l vs 15.3 ± 11.8 pmol/L, *P* < 0.001, *n* = 15–24) (Fig. 3C) and alkaline phosphatase (ALP) activities (*Sox3*^+/Y^ = 668.1 ± 161.9 U/L vs 258.7 ± 47.2 U/L, *P* < 0.001; *Sox3*^+/+^ = 820.8 ± 234.4 U/L vs 296.5 ± 58.4 U/L, *P* < 0.001, *n* = 16–25) (Fig. 3D). Wild-type mice on the low diet, compared to those on the control diet, also had significantly lower plasma urea concentrations (*Sox3*^+/Y^ = 5.98 ± 0.91 mmol/L vs 7.85 ± 1.24 mmol/L, *P* < 0.001; and *Sox3*^+/+^ = 5.69 ± 0.99 mmol/L vs 6.90 ± 1.13 mmol/L, *P* = 0.042, *n* = 18–25) (Fig. 3E) and creatinine concentrations (*Sox3*^+/Y^ = 14.3 ± 0.4 µmol/L vs 17.3 ± 0.8 µmol/L, *P* < 0.001; and *Sox3*^+/+^ = 14.9 ± 1.11 µmol/L vs 17.7 ± 0.9 µmol/L, *P* < 0.001, *n* = 17–23) (Fig. 3F).

Similar to wild-type mice, mutant male (*Sox3*^−/Y^) and heterozygote female (*Sox3*^+/^^−^) mice on the low diet also had significantly lower plasma corrected calcium concentrations than those on the control diet (*Sox3*^−/Y^ = 1.93 ± 0.20 mmol/L vs 3.05 ± 0.15 mmol/L, *P* < 0.001; and *Sox3*^+/^^−^ = 1.91 ± 0.14 mmol/L vs 3.06 ± 0.10 mmol/L, *P* < 0.001, *n* = 13–24) (Fig. 3B). This was associated with significantly higher plasma: PTH concentrations (*Sox3*^−/Y^ = 162.6 ± 37.6 pmol/L vs 12.8 ± 10.4 pmol/L, *P* < 0.001; and *Sox3*^+/^^−^ = 187.5 ± 28.6 pmol/L vs 9.9 ± 10.6 pmol/L, *P* < 0.001, *n* = 13–21) (Fig. 3C); and ALP activities (*Sox3*^−/Y^ = 700.3 ± 183.2 U/L vs 270.2 ± 49.4 U/L, *P* < 0.001; *Sox3*^+/^^−^= 906.4 ± 217.2 U/L vs 282.4 ± 48.4 U/L, *P* < 0.001, *n* = 12–22) (Fig. 3D). *Sox3*^−/Y^ and *Sox3*^+/^^−^ mice on the low diet also had significantly lower plasma: urea concentrations (*Sox3*^−/Y^ = 6.06 ± 0.92 mmol/L vs 7.83 ± 0.87 mmol/L, *P* < 0.001; and *Sox3*^+/^^−^ = 5.88 ± 0.92 mmol/L vs 7.10 ± 0.74 mmol/L, *P* < 0.018, *n* = 13–25) (Fig. 3E); and creatinine concentrations (*Sox3*^−/Y^ = 13.2 ± 1.3 µmol/L vs 16.8 ± 1.4 µmol/L, *P* < 0.001; and *Sox3*^+/^^−^ = 15.3 ± 1.3 µmol/L vs 17.7 ± 1.6 µmol/L, *P* < 0.002, *n* = 12–23) (Fig. 3F). The plasma corrected calcium and PTH concentrations in *Sox3*^−/Y^ and *Sox3*^+/^^−^ mice on the low diet were not statistically different to the wild-type mice on the low diet, indicating that *Sox3*^−/Y^ and *Sox3*^+/^^−^ mice have normal parathyroid function (Fig. 3B and C). Plasma ALP activities and urea and creatinine concentrations were also similar in wild-type mice compared to *Sox3*^−/Y^ and *Sox3*^+/^^−^ mice on the low calcium diet (Fig. 3D, E and F).

Histological analysis of parathyroid gland size from wild-type and mutant *Sox3* mice revealed that parathyroid gland areas in *Sox3*^+/Y^ and *Sox3*^−/Y^ mice were significantly greater, in keeping with diet-induced secondary hyperparathyroidism, in mice on the low diet, when compared to the control diet (*Sox3*^+/Y^ = 3885 ± 1012 µm^2^/g vs 1824 ± 656 µm^2^/g, *P* = 0.007; and *Sox3*^−/Y^ = 4672 ± 1914 µm^2^/g vs 2539 ± 684 µm^2^/g, *P* = 0.040, *n* = 4); however, there was no difference between *Sox3*^+/Y^ and *Sox3*^−/Y^ mice on each diet (normal diet *P* = 0.091; low diet *P* = 0.247) (Fig. 3G and H). Parathyroid gland sizes in *Sox3*^+/+^ and *Sox3*^+/^^−^ mice were similar on the low diet compared to the control diet (*Sox3*^+/+^ = 4065 ± 1178 µm^2^/g vs 2775 ± 1244 µm^2^/g, *P* = 0.091; and *Sox3*^+/^^−^ = 4542 ± 1664 µm^2^/g vs 2684 ± 1010 µm^2^/g, *P* = 0.052, *n* = 4), and there was no difference between *Sox3*^+/+^ and *Sox3*^+/^^−^ mice on each diet (normal diet *P* = 0.457; low diet *P* = 0.328) (Fig. 3G). Thus, *Sox3*^−/Y^ and *Sox3*^+/^^−^ mice appear to have normal parathyroid function and normal responses to hypocalcaemia, and loss of *Sox3* does not result in smaller parathyroids or HPT.

### Mice deleted for ultraconserved element* uc482* have normal plasma calcium and PTH concentrations

**Figure 4:**
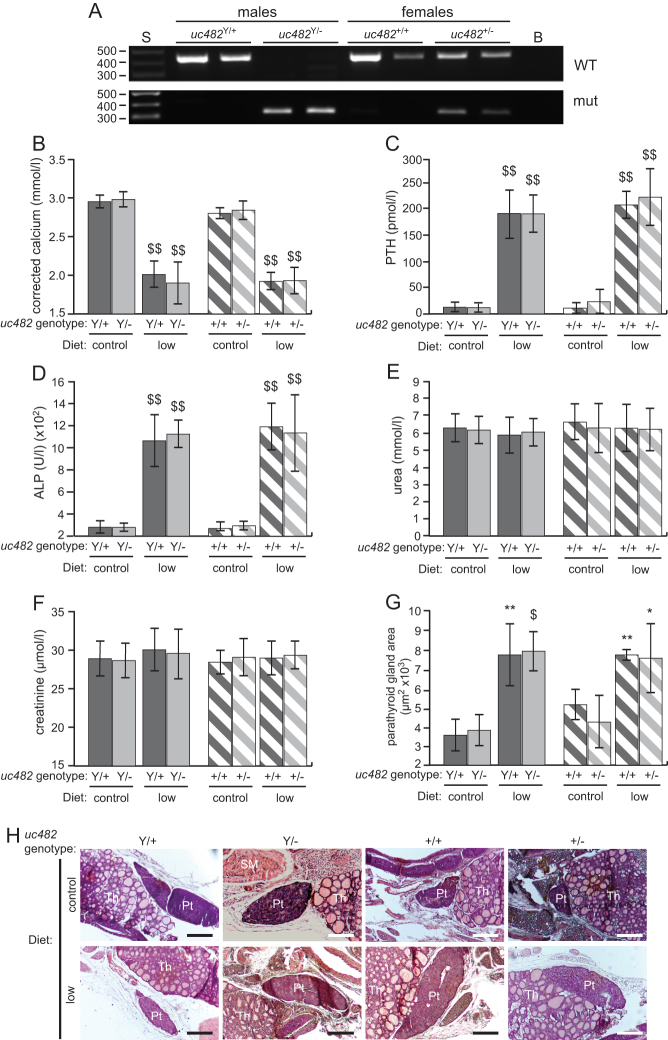
Plasma biochemistry and parathyroid gland sizes in wild-type and mutant *uc482* mice consuming either control or low calcium and vitamin D diets. (A) Genotyping of wild-type (*uc482*^+/Y^) and hemizygous (*uc482*^−/Y^) male, and wild-type (*uc482*^+/+^) and heterozygous (*uc482*^+/^^−^) female mice. S: size marker; B: blank; WT: wild-type band; mut: mutant band. (B) Plasma corrected calcium; (C) plasma PTH; (D) plasma alkaline phosphatase (ALP) activity; (E) plasma urea; (F) plasma creatinine; (G) parathyroid gland area. Wild-type males (+/Y) and mutant males (−/Y) are shown by the dark grey and light grey solid bars, respectively. Wild-type females (+/+) and mutant carrier females (+/−) are shown by the dark grey and light grey hatched bars, respectively. Data are presented as mean ± s.d. *P < 0.05; **P < 0.01; ^$^*P* < 0.001; ^$$^*P* < 0.0001 for mice on the low calcium and vitamin D (low Ca^2+^ and vit D) diet compared to mice of the same genotype on the normal diet. (H) Representative histological images used for quantification of parathyroid gland area. Pt: parathyroid; Th: thyroid; SM: strap muscle. Scale bars = 200 µm.

Mice were genotyped (Fig. 4A) and wild-type (*uc482*^+/Y^) and hemizygous (*uc482*^−/Y^) male adult mice, and wild-type (*uc482*^+/+^) and heterozygous (*uc482*^+/^^−^) female adult mice were found to have normal plasma biochemistry at the ages of 8 weeks and 28 weeks (Table 2). Thus, plasma corrected calcium concentrations were similar in *uc482*^+/Y^ mice compared to *uc482*^−/Y^ mice (2.95 ± 0.08 mmol/L vs 2.98 ± 0.10 mmol/L at 8 weeks, and 2.76 ± 0.08 mmol/L vs 2.83 ± 0.13 mmol/L at 28 weeks, *n* = 10−24); and in *uc482*^+/+^ mice compared to *uc482*^+/^^−^ mice (2.80 ± 0.07 mmol/L vs 2.84 ± 0.12 mmol/L at 8 weeks, and 2.70 ± 0.05 mmol/L vs 2.72 ± 0.12 mmol/L at 28 weeks, *n* = 7–42) (Table 2). Plasma PTH concentrations were also similar in *uc482*^+/Y^ and *uc482*^−/Y^ mice (12.9 ± 9.3 pmol/L vs 12.1 ± 9.0 pmol/L at 8 weeks, and 19.9 ± 16.7 pmol/L vs 26.7 ± 15.3 pmol/l at 28 weeks, *n* = 14–20); and in *uc482*^+/+^ and *uc482*^+/^^−^ mice (11.1 ± 10.4 pmol/L vs 23.5 ± 24.3 pmol/L at 8 weeks, and 18.4 ± 20.2 pmol/L vs 13.6 ± 12.0 pmol/L at 28 weeks, *n* = 9–19) (Table 2). In addition, the plasma phosphate, urea, creatinine and albumin concentrations, and alkaline phosphatase activities were similar in wild-type and mutant *uc482* mice at 8 weeks and 28 weeks of age (Table 2).

**Table 2:** Plasma biochemistry in 8 and 28 week old wild-type and mutant *uc482* mice consuming the control diet.

Genotype	Males		Females	
	*uc482*^+/Y^	*uc482*^−/Y^	*uc482*^+/+^	*uc482*^+/−^
Age: 8 weeks	(*n* = 17–25)	(*n* = 20–25)	(*n* = 9–15)	(*n* = 19–44)
Body weight (g)	21.32 ± 2.53	21.62 ± 1.49	17.51 ± 1.25	17.20 ± 1.51
Corr. Ca (mmol/L)	2.95 ± 0.08	2.98 ± 0.10	2.80 ± 0.07	2.84 ± 0.12
PTH (pmol/L)	12.9 ± 9.3	12.1 ± 9.0	11.1 ± 10.4	23.5 ± 24.3
Phosphate (mmol/L)	4.10 ± 0.70	4.17 ± 0.39	4.01 ± 0.54	3.95 ± 0.52
ALP (U/L)	280.7 ± 53.9	279.8 ± 36.8	286.8 ± 41.1	293.8 ± 40.1
Urea (mmol/L)	6.29 ± 0.80	6.16 ± 0.79	6.63 ± 1.04	6.29 ± 1.42
Creatinine (µmol/L)	28.88 ± 2.23	28.63 ± 2.20	28.43 ± 1.55	29.05 ± 2.38
Albumin (g/L)	29.8 ± 1.5	29.0 ± 1.3	32.0 ± 1.9	31.7 ± 1.5
Age: 28 weeks	(*n* = 8–14)	(*n* = 10–16)	(*n* = 11–15)	(*n* = 8–12)
Body weight (g)	37.13 ± 4.94	37.79 ± 5.28	27.07 ± 4.93	26.14 ± 4.28
Corr. Ca (mmol/L)	2.76 ± 0.08	2.83 ± 0.13	2.70 ± 0.05	2.72 ± 0.12
PTH (pmol/L)	19.9 ± 16.7	26.7 ± 15.3	18.4 ± 20.2	13.6 ± 12.0
Phosphate (mmol/L)	3.55 ± 0.55	3.71 ± 0.66	3.20 ± 0.54	3.42 ± 0.51
ALP (U/L)	69.7 ± 7.5	71.0 ± 7.0	79.7 ± 8.5	85.3 ± 18.8
Urea (mmol/L)	10.04 ± 1.04	9.23 ± 1.21	8.46 ± 0.83	8.73 ± 1.18
Creatinine (µmol/L)	33.8 ± 2.2	35.3 ± 2.2	33.4 ± 1.7	34.3 ± 3.0
Albumin (g/L)	30.5 ± 1.3	31.1 ± 1.3	31.5 ± 1.8	31.8 ± 1.6

Mean ± s.d. values are shown. Wild-type males (uc482^+/Y^), mutant males (uc482^−/Y^), wild-type females (uc482^+/+^) and mutant carrier females (uc482^+/^^−^). There were no significant differences between knock-out and wild-type littermates at the same age.

ALP, alkaline phosphatase; Corr. Ca, corrected calcium; PTH, parathyroid hormone.

When challenged with the low diet for 60 days, plasma corrected calcium concentrations were significantly lower in: male wild-type (*uc482*^+/Y^) and mutant hemizygous (*uc482*^−/Y^) mice (*uc482*^+/Y^ = 2.01 ± 0.17 mmol/L vs 2.95 ± 0.08 mmol/L, *P* < 0.0001; and *uc482*^−/Y^ = 1.90 ± 0.27 vs 2.98 ± 0.10 mmol/L, *P* < 0.0001, *n* = 10–24); and in female wild-type (*uc482*^+/+^) and heterozygous (*uc482*^+/^^−^) mice (*uc482*^+/+^ = 1.92 ± 0.11 mmol/L vs 2.80 ± 0.07 mmol/L, *P* < 0.0001; and *uc482*^+/^^−^ = 1.93 ± 0.17 mmol/L vs 2.84 ± 0.12 mmol/L, *P* < 0.0001, *n* = 14-42) (Fig. 4B). These lower plasma corrected calcium concentrations were not due to lower plasma albumin concentrations when on the low diets, as these plasma albumin concentrations were similar (uc482^+/+^ = 30.44 ± 0.34 g/L, uc482^+/-^ = 30.73 ± 0.26 g/L, uc482^+/Y^ = 30.63 ± 0.38 g/L and uc482^−/Y^ = 30.41 ± 0.70 g/L), on low diet to those observed in mice on the control diet (Table 2). This was associated with significantly higher plasma PTH concentrations in *uc482*^+/Y^ and *uc482*^−/Y^ mice (*uc482*^+/Y^ = 194.6 ± 48.2 pmol/L vs 12.9 ± 9.3 pmol/L, *P* < 0.0001; and *uc482*^−/Y^ = 194.0 ± 36.3 pmol/L vs 12.1 ± 9.0 pmol/L, *P* < 0.0001, *n* = 10–20); and in *uc482*^+/+^ and *uc482*^+/^^–^ mice (*uc482*^+/+^ = 211.3 ± 26.4 pmol/L vs 11.1 ± 10.4 pmol/L, *P* < 0.0.0001; and *uc482*^+/^^–^ = 226.3 ± 54.4 pmol/L vs 23.5 ± 24.3 pmol/L, *P* < 0.0001, *n* = 9–19) (Fig. 4C). Plasma ALP activities were also significantly higher in: *uc482*^+/Y^ and *uc482*^−/Y^ mice (*uc482*^+/Y^ = 1062.7 ± 233.1 U/L vs 280.7 ± 53.9 U/L, *P* < 0.0001; and *uc482*^−/Y^ = 1123.0 ± 125.4 U/L vs 279.8 ± 36.8 U/L, *P* < 0.0001, *n* = 17–29); and in *uc482*^+/+^ and *uc482*^+/^^–^ mice (*uc482*^+/+^ = 1189.9 ± 211.0 U/L vs 286.8 ± 41.1 U/L, *P* < 0.0001; and *uc482*^+/^^−^ = 1133.7 ± 346.5 U/L vs 293.8 ± 40.1 U/L, *P* < 0.0001, *n* = 13–40) (Fig. 4D). The plasma corrected calcium and PTH concentrations, and plasma ALP activities in *uc482*^–/Y^ and *uc482*^+/^^–^ mice on the low diet were similar to those of the *uc482*^+/Y^ and *uc482*^+/+^ mice, respectively, on the control diet, indicating that *uc482*^−/Y^ and *uc482*^+/^^−^ mice have normal parathyroid function (Fig. 4B, C and D). There were no differences in plasma urea or creatinine concentrations between *uc482^+/Y^*, *uc482^+/+^*, *uc482^−/Y^* or *uc482^+/^*^−^ mice on the low diet compared to the control diet (Fig. 4E and F).

Histological analysis of parathyroid gland size from wild-type and mutant *uc482* mice revealed that parathyroid gland areas in all genotypes were significantly larger, in keeping with diet-induced secondary hyperparathyroidism, in mice on the low diet compared to the control diet (*uc482*^+/Y^ = 7697 ± 1596 µm^2^/g vs 3545 ± 824 µm^2^/g, *P* = 0.002; *uc482*^−/Y^ = 7887 ± 1016 µm^2^/g vs 3797 ± 802 µm^2^/g, *P* < 0.001; *uc482*^+/+^ = 7525 ± 1780 µm^2^/g vs 4220 ± 1348 µm^2^/g, *P* = 0.013; and *uc482*^+/^^−^ = 7693 ± 276 µm^2^/g vs 5123 ± 782 µm^2^/g, *P* = 0.004, *n* = 4) (Fig. 4G, H). There were no differences between *uc482*^+/Y^ and *uc482*^−/Y^ mice, or between *uc482*^+/+^ and *uc482*^+/^^−^ mice, either on the control diet, or the low diet (*uc482*^+/Y^ vs *uc482*^−/Y^ control diet *P* = 0.338 and low diet *P* = 0.424; and *uc482*^+/+^ vs *uc482*^+/^^−^ control diet *P* = 0.145 and low diet *P* = 0.432). Thus, *uc482*^−/Y^ and *uc482*^+/^^−^ mice appear to have normal parathyroid gland function and normal responses to hypocalcaemia, and loss of *uc482* does not result in smaller parathyroids or HPT.

## Discussion

**Figure 5:**
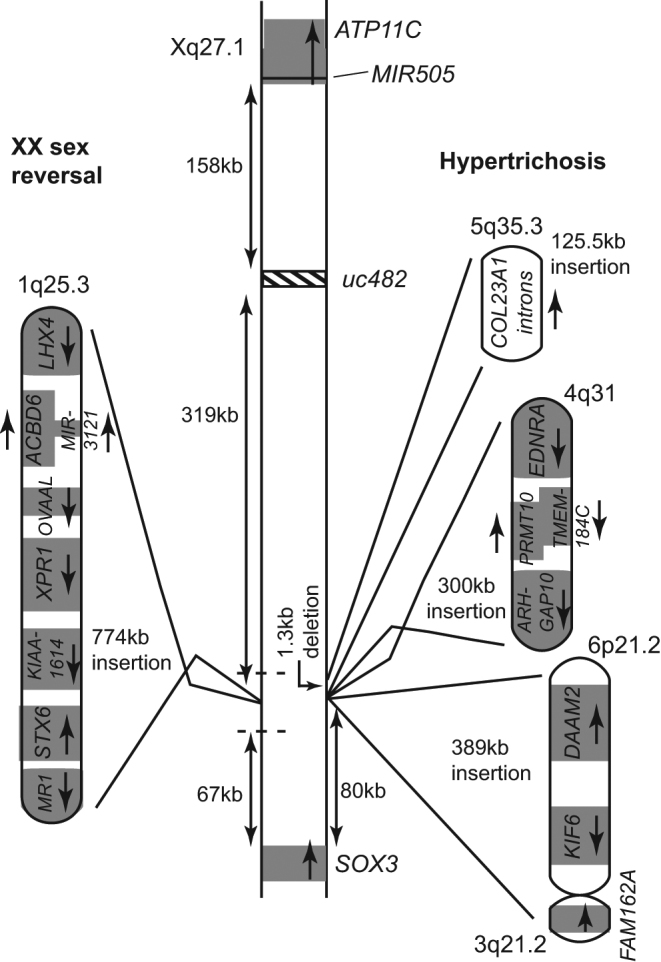
Chromosomal rearrangements associated with XX sex reversal and hypertrichosis. A patient with XX sex reversal was found to have an interstitial deletion-insertion involving chromosome Xq27.1 and chromosome 1q25.3 (11). One family from China and two unrelated families from Mexico with hypertrichosis have been reported to have interstitial deletion-insertions involving chromosome Xq27.1 and chromosomes 5q35.3, 4q31, and 6p21.2, respectively (23, 24, 25). Dotted lines denote the location of the XLHPT interstitial deletion-insertions.

The molecular and cellular effects of the interstitial deletion-insertion involving chromosomes Xq27.1 and 2p25.3 in two families with XLHPT and their effects on *SOX3* expression in the etiology of HPT remain to be elucidated (Fig. 1). Elucidation of the mechanism(s) may also have relevance to other disorders, as interstitial deletion-insertions involving deletions at the same locus on Xq27.1, but insertions from other autosomes, have been described in other diseases (7, 24, 25, 26) (Fig. 5). For example, three families with X-linked congenital generalized hypertrichosis (CGH) have been reported to have deletion-insertions comprising: a Chinese family with a ~1.3 kb Xq27.1 deletion and a ~125.5 kb 5q35.3 insertion containing intronic sequence from within collagen type 23, alpha 1 (*COL23A1*); a Mexican family with a 7 bp deletion and a ~300 kb 4q31.22-31.23 inverted insertion containing protein arginine methyltransferase 10 (*PRMT10*), transmembrane protein 184C (*TMEM184C*), and parts of Rho GTPase activating protein 10 (*ARHGAP10*) and endothelin receptor type A (*EDNRA*); and another Mexican family with a 2 bp Xq27.1 deletion and a ~389 kb 6p21.2 insertion containing Dishevelled-associated activator of morphogenesis 2 (*DAAM2)* and kinesin family member 6 (*KIF6*) and a 56 bp 3q21.1 insertion containing part of family with sequence similarity 162 member A (*FAM162A*) (27, 28, 29) (Fig. 5). There are two distinguishing clinical features in these CGH families. In the Chinese family, the presence of spina bifida in the proband and scoliosis in 4 family members implicates a role for *SOX3*, which has been linked to neural tube defects in man (24, 25), chicken (26), Drosophila (30) and zebrafish (31). In the Mexican family with the 6p21.2 and 3q21.1 insertions, several members also had dental anomalies. The disease mechanism in this family was postulated to be a position effect on fibroblast growth factor 13 (*FGF13*), located ~1.2 Mb proximal to the insertion site, and decreased expression of *FGF13*, which is found in developing hair follicles, dental mesenchyme and in developing tooth buds, was detected in patient keratinocytes (27). By contrast, a patient with XX male sex reversal was found to have a 4 bp deletion at the same locus on Xq27.1 and a ~774 kb 1q25.2-25.3 insertion containing 6 complete genes (acyl-CoA binding domain containing 6 (*ACBD6*), xenotropic and polytropic retrovirus receptor 1 (*XPR1*), unknown gene *KIAA1614*, syntaxin 6 (*STX6*), ovarian adenocarcinoma amplified long ncRNA (*OVAAL*), and microRNA 3121 (*MIR3121*)) and two partial genes (LIM homeobox 4 (*LHX4*) and major histocompatibility complex, class-I related (*MR1*)), that switched on *SOX3* expression in lymphoblastoid cells from the patient but did not affect *FGF13* expression (12) (Fig. 5). Combined, these studies demonstrate that diverse phenotypes can result from similar sized insertions into the same Xq27.1 locus, and that similar phenotypes (e.g. CGH) can result from insertions from different autosomes, highlighting the complexity underlying long-range gene regulation and the effects of chromosomal rearrangements in disease. Indeed, Hi-C chromatin interaction analyses have revealed that the alterations of genomic repressors or enhancers can affect long-range gene expression, and thus it is feasible that these deletion-insertions of Xq27.1 may affect genes that are nearby *SOX3*, but which exert their effects on different distant genes. For example, *MIR505* located downstream of *SOX3* (Figs 1 and 5) encodes a microRNA that inhibits: neural tube formation by targeting fibroblast growth factor 18 (*FGF18*) located on chromosome 5q35.1 (32); and cell growth and endothelial migration by targeting mitogen-activated protein kinase kinase kinase 3 (MAP3K3) through the AKT-NF-κB pathway whose genes are located on chromosomes 17q23.3 (*MAP3K3*), 14q32.33 (AKT serine/threonine kinase 1 (*AKT*)), 10q24.31 (component of inhibitor of nuclear factor kappa B kinase complex (*IKK1*)), 8p11.21 (inhibitor of nuclear factor kappa B kinase subunit beta (*IKK2*)), 4q24 (nuclear factor kappa b subunit 1 (*NFKB1*)), and 11q13.1 (RELA proto-oncogene, NF-KB subunit (*RELA*)) (33). Thus, altered expression of *MIR505* could be involved in the occurrence of spina bifida in the proband from the Chinese family with CGH that is associated with a deletion of Xq27.1 and insertion of 5q35.3 (29), although it also remains possible that it is the deletion-insertion associated disruption of *SOX3* itself that is responsible for spina bifida, as SOX3 dysfunction has been reported to be linked to neural tube defects in human (24, 25). Different mechanisms are likely to be involved in the phenotypes of the other CGH families with insertions of 4q31.22-31.23 and 6p21.2 (27, 28), and the XX male sex reversal patient, with insertion of 1q25.2-25.3, who did not have neural tube defects (12). Thus, it seems that different types of insertions in the same locus can affect expression in other genes and thereby result in different phenotypes. Plasma biochemistry has not been reported in CGH or XX male sex reversal, and thus any co-occurrence of HPT and associated hypocalcaemia in these patients remains unknown, although such occurrence of HPT in these patients would seem unlikely as hypocalcaemic seizures are likely to have been recognized and also as XLHPT patients do not have hypertrichosis or XX male sex reversal.

In the two XLHPT families, the two deletions within Xq27.1 are of different sizes but do overlap, whilst the two insertions which both originate from 2p25.3 do not overlap, and both deletion-insertions lead to a net gain of DNA of differing amounts (~315 kb and ~46 kb), resulting in the same phenotype. This comparison may help to elucidate the mechanism(s) by which the deletion-insertion alters *SOX3* expression (Fig. 1B). First, this may be due to loss of an enhancer or repressor from the shared missing segment of the X chromosome. This is possible because there is overlap such that the 1.5 kb deletion in the IL family is contained within the 25 kb deletion from the MO family, but unlikely because the small 1.5 kb region does not contain any ultraconserved sequences. Second, this may be due to gain of an enhancer or repressor within the inserted DNA. This is possible because most of the inserted DNA is non-coding, but unlikely because the two inserted regions in the two families do not overlap, and there is a low probability that two different insertions would both result in the same subtle effect on timing of *SOX3* expression in the developing parathyroid glands, as is postulated in these two families. Third, the deletion-insertion may move an enhancer or repressor away from *SOX3*. This is possible because both deletion-insertions cause net gain of DNA. One such regulatory element, which has been shown to be a likely enhancer of gene expression in zebrafish, is *uc482* (16), although *uc482* has not been shown directly to affect *SOX3* expression and our studies reveal that mice lacking *uc482* do not develop HPT (Fig. 4 and Table 2). However, there are also 4 other ultraconserved elements in the region between *SOX3* and *uc482*, all of which would also be affected by the interstitial deletion-insertions (16), so a combinatorial loss of these elements may be required to alter SOX3 expression and cause HPT. Finally, the deletion-insertion may disrupt a topological associated domain (TAD), which represents a fundamental three-dimensional evolutionarily conserved genomic structural unit that directs regulatory elements to their promoters and insulates them from other genes (34, 35). Examination for the locations of TAD boundaries on the X chromosome in human non-parathyroid cell types using Hi-C chromatin interaction data did not identify TAD boundaries between the *SOX3* and *ATP11C* genes, with the nearest TAD boundary being located approximately 135 kb upstream of *SOX3* (36). It is therefore unlikely that the deletion/insertion involving chromosomes Xq27.1 and 2p25.3 altered an existing TAD boundary on the X chromosome, although the possibility that the inserted chromatin may have resulted in the generation of a new TAD boundary to alter *SOX3* expression cannot be excluded.

*SOX3* expression was not altered by the Xq27.1/2p25.3 deletion-insertion in EBV-lymphoblastoid cells from our XLHPT patients (Fig. 2), and this may or may not reflect the situation in developing parathyroid cells. However, absence of *Sox3* expression that would occur in parathyroids of mutant mice with deletion of *Sox3* did not result in hypocalcemia or HPT in *Sox3*^-/Y^ mice (Fig. 3 and Table 1). Thus, this result indicates that complete loss of *Sox3* expression is unlikely to cause HPT, and that XLHPT is likely to be due to a more complex mechanism.

Nevertheless, a role for SOX3 in parathyroid development is highly likely as *Sox3* expression has been detected in developing mouse parathyroid glands between E10.5 and E15.5 (8). However, the absence of any decrease in parathyroid size or development of HPT in mice lacking *Sox3*, even when challenged with a low calcium diet lacking vitamin D, indicates that loss of *Sox3* alone is not sufficient to cause HPT and that other SOX family transcription factors may compensate for loss of SOX3. Indeed, SOX proteins, which are arranged into groups designated A-H, compensate for each other within the same group. For example, two members of the SOXF group, SOX17 and SOX18, compensate for each other in developing vascular endothelial cells (37). SOX3 is a member of the SOXB1 group, which also includes SOX1 and SOX2, and all three members share significant protein sequence homology, which is at its greatest (~95%) within the high mobility group (HMG) DNA-binding domain (38). Furthermore, these SOXB1 members have overlapping expression patterns in the CNS (38), and despite expression of *Sox3* throughout the developing CNS, *Sox3* null mice develop specific defects only in the hippocampus, corpus callosum and hypothalamus, and it is therefore postulated that partial compensation by SOX1 and/or SOX2 may prevent a more severe neurological phenotype in *Sox3* null mice (19, 39). Indeed, redundancy between SOX2 and SOX3 has been shown for morphogenesis of the second pharyngeal arch during development in mice (39). The related SOXB1 factor, SOX19B, also acts redundantly with SOX3 in restricting WNT signalling to the organiser during CNS development and dorsoventral patterning (40).

The absence of HPT in *Sox3*^−/Y^ and *Sox3*^+/^^−^ mice indicates that loss of *Sox3* expression is not the underlying mechanism in XLHPT. Altered expression of *Sox3* in three other ways could potentially be considered as possible mechanisms for XLHPT. First, the deletion-insertions may result in *Sox3* overexpression. However, this seems unlikely, as patients with *SOX3* duplications have been reported to have hypopituitarism, mental retardation, and XX male sex reversal, which are not features of XLHPT (24, 41, 42). The other two possible mechanisms are that *Sox3* could either be expressed inappropriately early in the developing pharyngeal pouches, or that there might be inappropriate continued expression after the window in which *Sox3* would normally be expressed. *Sox3* expression in the mouse is detected in developing parathyroid glands at E10.5, E13.5, and E15.5, but not at E18.5, indicating that the timing of *Sox3* expression is likely to be important, as has been shown for other SOX proteins during organogenesis. For example, SRY expression in somatic cells of the male genital ridge has a critical expression window of only ~6 hours, during which its expression must reach a certain threshold level to trigger Sertoli cell differentiation (43). Consistent with this hypothesis, we could not demonstrate altered *SOX3* expression in lymphoblastoid cells from XLHPT patients, which might not be representative of SOX3 time and/or tissue specific expression, as for example in the parathyroid glands. Investigation of a temporospatial effect on *SOX3* expression in developing parathyroid glands is not possible in XLHPT patients, and will require a mouse model engineered to harbour the appropriate chromosomal rearrangements.

The lower plasma urea and creatinine concentrations in the wild-type and mutant *Sox3* mice, which are on a MF1 outbred background when on the low calcium and vitamin D diet (low diet) is interesting and likely to be a strain specific response to this dietary challenge. Thus, the plasma urea and creatinine concentrations did not decrease in response to the challenge with the low calcium and vitamin D diet, in the wild-type and mutant *uc482* mice (Fig. 4E and F), which are on a C57Bl/6 background, and in previously reported wild-type and mutant *Gata3* mice, which were on a FVB/N background, (21). The findings of a decreased plasma urea and creatinine concentrations in both wild-type and mutant *Sox3* mice, but not in wild-type or mutant *uc482* and *Gata3* mice, indicate that these decreased plasma concentrations are not mutant dependent, but instead are strain-specific and may involve modifier genes that influence urea and creatinine metabolism. In addition, the observed changes in creatinine metabolism due to low vitamin D in the MF1 strain (Fig. 3) are similar to that reported in healthy populations (without chronic kidney diseases) from Taiwan and Spain, in whom low dietary vitamin D was found to correlate with low plasma creatinine concentrations (44, 45).

In conclusion, XLHPT does not result from the absence of expression of *SOX3* or absence of the regulatory element *uc482*. Instead, the interstitial deletion-insertions likely have other effects on the temporospatial expression of *SOX3* in the developing parathyroid glands.

## Declaration of interest

The authors declare that there is no conflict of interest that could be perceived as prejudicing the impartiality of the research reported.

## Funding

This work was supported by the Medical Research Council, UK (grant numbers G9825289 and G1000467), MRC PhD Studentships (K U G, S M M), Wellcome Trust Senior Investigator Award (R V T), Cancer Research UK, the Wellcome Trust (grant number FC001107), and Shriners Hospitals for Children.

## Author contribution statement

Study design: R L B, R V T; study conduct and data collection: K U G, I V G, S M M, S E P, K G K, M S, M R B, M A N, P T C, B F, T H; data analysis and interpretation: K U G, I V G, S M M, S E P, K G K, M S, K R, M R B, M A N, B F, T H, M P W, R L B, R V T; drafting manuscript: K U G, S E P, R V T; revising manuscript content and approving final version of the manuscript: K U G, I V G, S M M, S E P, K G K, M S, K R, M R B, M A N, P T C, B F, T H, M P W, R L B, R V T. R V T takes responsibility for the integrity of the data analysis.
